# Do Actomyosin Single-Molecule Mechanics Data Predict Mechanics of Contracting Muscle?

**DOI:** 10.3390/ijms19071863

**Published:** 2018-06-25

**Authors:** Alf Månsson, Marko Ušaj, Luisa Moretto, Dilson E. Rassier

**Affiliations:** 1 Department Chemistry Biomedical Sciences, Linnaeus University, SE-39182 Kalmar, Sweden; marko.usaj@lnu.se (M.U.); luisa.moretto@lnu.se (L.M.); 2 Department Kinesiology and Physical Education, McGill University, Montreal, QC H3A 2T5, Canada

**Keywords:** optical tweezers, optical traps, muscle fiber, myofibril, myosin, actin, cross-bridge, mechanochemical model

## Abstract

In muscle, but not in single-molecule mechanics studies, actin, myosin and accessory proteins are incorporated into a highly ordered myofilament lattice. In view of this difference we compare results from single-molecule studies and muscle mechanics and analyze to what degree data from the two types of studies agree with each other. There is reasonable correspondence in estimates of the cross-bridge power-stroke distance (7–13 nm), cross-bridge stiffness (~2 pN/nm) and average isometric force per cross-bridge (6–9 pN). Furthermore, models defined on the basis of single-molecule mechanics and solution biochemistry give good fits to experimental data from muscle. This suggests that the ordered myofilament lattice, accessory proteins and emergent effects of the sarcomere organization have only minor modulatory roles. However, such factors may be of greater importance under e.g., disease conditions. We also identify areas where single-molecule and muscle data are conflicting: (1) whether force generation is an Eyring or Kramers process with just one major power-stroke or several sub-strokes; (2) whether the myofilaments and the cross-bridges have Hookean or non-linear elasticity; (3) if individual myosin heads slip between actin sites under certain conditions, e.g., in lengthening; or (4) if the two heads of myosin cooperate.

## 1. Introduction

**Figure 1:**
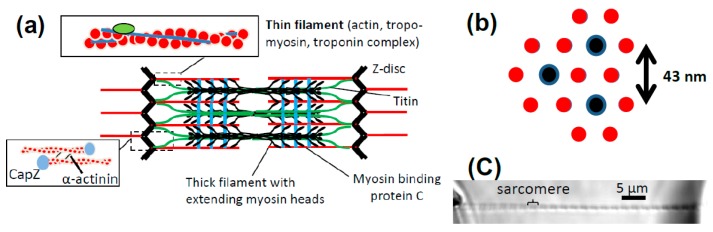
Myofibrils and the functional unit of muscle. (**a**) Schematic of one sarcomere (region between two Z-discs; length ~2 μm) with key protein components; (**b**) cross-section of the overlap region between actin and myosin in the sarcomere showing each thick filament surrounded by six thin filaments with and overall 2:1 ratio between the number of thin filaments and thick filaments. Same color coding as in (**a**)*.* Center-to-center distance of thick filaments from [10]; (**c**) myofibril isolated from rabbit psoas muscle and mounted for mechanical experiments.

Contraction of striated muscle is the result of adenosine triphosphate (ATP)-driven cyclic interactions between the contractile proteins myosin II (often “myosin” below) and actin (Figure 1). The actin-containing thin filaments and the myosin-containing thick filaments in the sarcomere (Figure 1a) are organized into a nearly crystalline hexagonal lattice [1] where each thick filament is surrounded by 6 thin filaments (Figure 1b). In addition to actin and myosin, the filaments contain a range of accessory proteins, often with regulatory and modulatory roles, such as troponin and tropomyosin in the thin filaments [2,3], myosin-binding protein C (MyBPC) in the thick filaments [4,5] and the elastic protein titin [6,7] spanning the thin and thick filaments (Figure 1a). The latter MDalton protein contributes both to the mechanical stability of the sarcomere and plays roles in mechano-sensing [8,9].

The half-sarcomere is the functional unit of muscle containing virtually complete contractile and regulatory machinery. The self-assembly of multiprotein filaments into the highly ordered myofilament lattice [11] is essential for the effective operation of the half-sarcomere but this ordered arrangement is necessarily lost in single-molecule mechanics studies. On the next hierarchical level, the half-sarcomeres are serially interconnected to form a myofibril of 1–2 μm diameter. Furthermore, the myofibrils are arranged in parallel with the sarcomere pattern largely in register over the muscle fiber cross-section due to interconnections via intermediary filaments such as desmin between sarcomere Z-lines of neighboring myofibrils. This lateral register, or the interconnecting desmin links per se, is of significance for optimal function [12]. On yet higher organizational level, the muscle cells form bundles with different arrangements relative to the tendons to either optimize for high maximum force or high maximum velocity [13]. On the highest level, several muscles work together in a well-coordinated geometrical arrangement to achieve effective and smooth motion around one or several joints. The geometrical arrangements on different levels have important implications for overall contractile function. On an intermediate level, the muscle cells or rather the motor units (one motor neuron and all cells innervated by this neuron) are the building blocks that the nervous system uses for producing complex motions. The present account is limited to the issue of how mechanical properties of a contracting cell can be predicted by single-molecule mechanics data. The higher hierarchical levels are not further considered.

The sarcomere arrangement with actin and myosin in interdigitating filaments is clearly of great value to survival as it has evolved independently in the phylogenetically distant bilaterians (e.g., mammals) and cnidarians (e.g., jellyfish) [14]. One obvious benefit is that serial connection of half-sarcomeres enables effective summation of velocities and length changes. This allows the molecular size structural changes of the motor to produce large-scale bodily motions. Specifically, the 5–10 nm length change produced by the stroke of each myosin motor is, by cyclic operation of a team of motors, translated into a maximum sliding velocity of 20 μm/s between the thin and the thick filaments in a half-sarcomere. In turn, this velocity, impressive in itself compared to molecular dimensions, is transformed into a velocity of almost 2 m/s of 10 cm long muscle cell with ~100,000 half-sarcomeres in series. In addition, parallel arrangement of all half-sarcomeres over the cross-section of the muscle cells enables effective summation of the forces produced by all myosin cross-bridges. Together with special arrangements of the muscle fibers relative to the tendons, this enables development of impressive maximum forces by a given muscle, such as 15 N (1.5 kg) for the extensor tibiae muscles [15] of tiny grasshoppers where the muscle itself weighs less than 100 mg. The developed force reflects the simultaneous action of about 3 × 10^12^ myosin cross-bridges per half-sarcomere over the muscle cross-sectional area with each myosin head developing a force of approximately 5 pN.

The hierarchically ordered structure of muscle cells ideally means that whole cell displacements correspond directly to nm displacements of the myosin motors in each half-sarcomere. Furthermore, the total force developed by a muscle cell would be the sum of the forces produced by all cross-bridges per half-sarcomere over the muscle fiber cross-section. These are basic assumptions made in mechanical experiments on muscle cells and myofibrils to enable interpretation of the results in terms of cross-bridge properties. The extent to which these assumptions are true is of critical importance for the interpretation of a range of muscle mechanical experiments. Furthermore, the degree to which single-molecule results and muscle properties show direct correspondence is of interest for drug discovery efforts with myosin II in focus [16,17,18,19,20,21,22,23,24,25,26,27,28,29] as well as for efforts to understand molecular mechanisms in sarcomere myopathies, e.g., cardiomyopathies, due to mutations in sarcomere proteins [29,30,31,32,33,34]. Thus, several of these studies use in vitro assays either of single molecules or small disordered actin and myosin ensembles. In this connection, however, it is important to consider the possibility that emergent phenomena [35] as well as accessory regulatory proteins [5,8,30,31,32,36,37,38] have increasing influence in diseases compared to physiological conditions.

In the present review, we first summarize key results from both single-molecule mechanics experiments and muscle or myofibril mechanics studies and consider different challenges associated with these different experimental systems. We then analyze to what degree the resulting views of the mechanisms of muscle contraction and the cross-bridge properties that emerge from the different approaches, are consistent with each other. We find very good agreement in several respects but also identify several poorly understood issues.

## 2. Mechanical Measurements on Isolated Molecular Motors—Principles and Basic Findings

**Figure 2:**
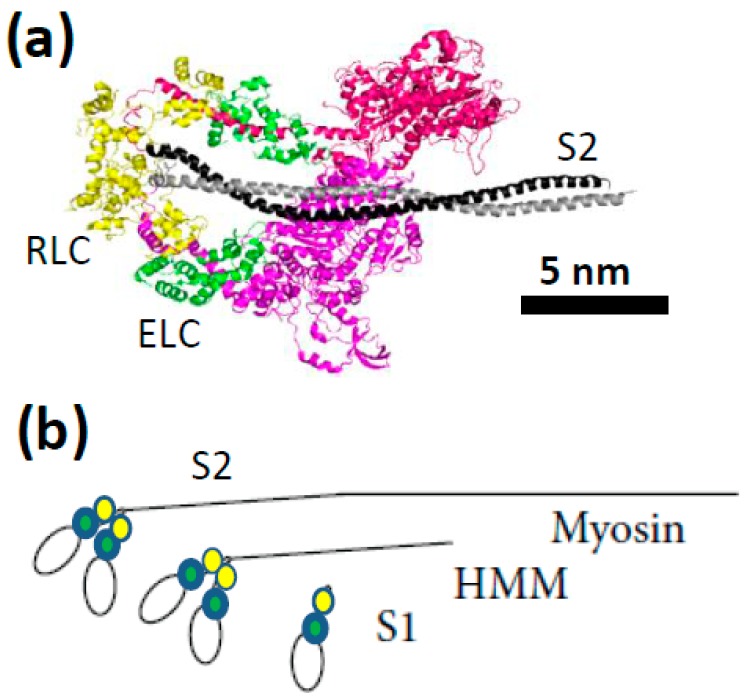
The myosin molecule and some key proteolytic fragments. (**a**) Crystal structure (PDB: 3DTP) showing two myosin motor domains with regulatory (yellow; RLC) and essential (green; ELC) light chains and a part of the sub-fragment 2 (S2) tail (black) of the myosin heavy chain. The construct is shown in the interacting head configuration believed to correspond to the super-relaxed state in muscle cells [40,41]; (**b**) schematic illustration of full length myosin and two soluble proteolytic fragments, heavy meromyosin (HMM) and sub-fragment 1 (S1) that are often used in single-molecule mechanics.

Myosin II of muscle is a heterohexameric protein composed of two heavy chains, two regulatory light chains and two essential light chains (Figure 2a). The C-terminal of the myosin heavy chain is alpha-helical allowing two heavy chains to form a coiled coil tail. This tail both holds the two heavy chains together into one myosin molecule and interacts with other myosin tails to form the backbone of the thick filament within a sarcomere. The tail region of each heavy chain is connected via an alpha-helical neck region (the lever arm) to a globular motor domain with actin-binding and catalytic sites at the N-terminus of the heavy chain. The neck region is stabilized by one regulatory light chain (RLC) and one essential light chain (ELC). The detailed molecular structure of myosin and the relation to motor function is considered in recent reviews [30,39].

**Figure 3:**
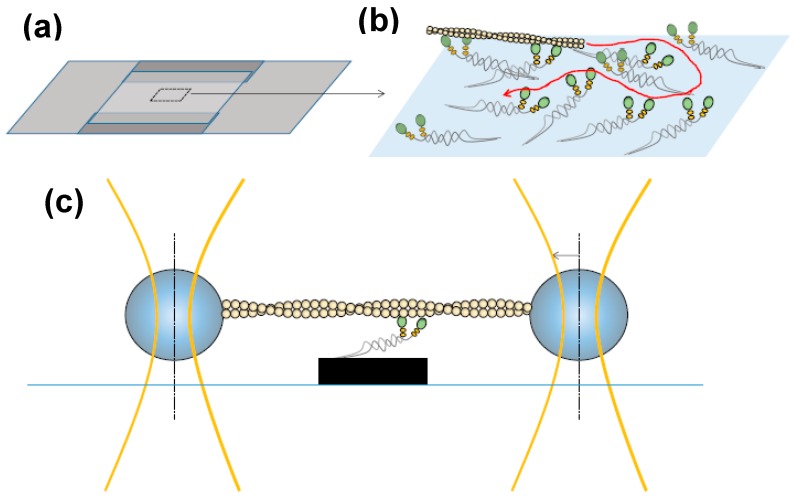
In vitro motility assay and optical tweezers set-up for single-molecule mechanics. (**a**) Flow cell of two cover-slips sandwiched on top of each other via spacers; (**b**) magnified view of rectangular area in (**a**) indicating the principle for the gliding in vitro motility assay with surface-adsorbed myosins that propel an actin filaments. Curved arrow (red) indicates possible filament sliding path; (**c**) schematic of the three-bead optical tweezers assay where an actin filament is suspended between two beads held in optical traps. The filament is then lowered down to allow the actin filament to interact with single myosin motor fragments adsorbed to a third bead or another type of pedestal as indicated here.

In experiments on isolated proteins, soluble proteolytic [42,43] or expressed myosin fragments [38,44,45,46,47,48] are usually used (Figure 2b) such as one-headed sub-fragment 1 (S1; of varying lengths) or two-headed heavy meromyosin (HMM). The latter fragment may be obtained by chymotryptic cleavage of full length myosin and contains two S1-domains and the, so called, sub-fragment 2 (S2) part of the myosin tail (Figure 2b). A HMM construct of human cardiac myosin has also recently been expressed and purified [38] from mouse myotubes. The development of in vitro motility assays (Figure 3) in the 1980s [49,50,51] opened for studies of mechanical properties of isolated myosin and actin molecules. In the most frequently used version of this assay (“the gliding assay”), the propulsion of fluorescence labeled actin filaments in the absence of external load is observed as the actin filaments are propelled by surface-adsorbed myosin molecules or, more commonly, the proteolytic motor fragments HMM or S1 (Figure 2b). Soon after development of the in vitro motility assay, force measurements were performed using microfabricated custom-made cantilevers applied to an ensemble (<100) of myosin motor domains [52]. These experiments conclusively showed that one S1 motor domain is sufficient for force production. As the next step towards single-molecule mechanics, in vitro motility assays were combined with optical tweezers [53,54,55,56], with improved force sensitivity compared to cantilevers. In the mid-1990s studies, using this approach [57], discrete motor steps of around 10 nm for a single myosin motor and average forces per motor of about 5 pN were convincingly demonstrated.

The three-bead optical tweezers assay (Figure 3c) was developed by Spudich and co-workers [57] for studies of muscle myosin. In this configuration, an actin filament is mounted between two micrometer sized beads (in a dumbbell configuration) each bead held in a separate optical trap (Figure 3). This is followed by lowering the filament down to a third bead, or pedestal, sparsely coated with myosin motors. Such an arrangement where the actin filament is held between two beads is necessary for single-molecule studies of myosin II to prevent diffusion of the interacting partners away from each other due to the low fraction of the ATP-turnover time that myosin is actually bound to actin. The dumbbell arrangement has been the basis for virtually all later optical tweezers-based studies of the actin-myosin motor system and other, so called, non-processive motors that only make brief encounters with their tracks [47,58,59,60,61,62,63,64,65,66,67]. Also a large fraction of other studies of biomolecular function using optical tweezers have employed this arrangement because it reduces important sources of noise by uncoupling the system from the microscope stage [68,69,70,71]. The developments have been reviewed in detail elsewhere [72,73].

Structural studies e.g., using cryo-electron microscopy or X-ray crystallography give increasingly detailed snap-shots of specific stable or metastable myosin or actomyosin states [39]. However, only functional assays (e.g., using single-molecule measurements of force-and displacement) can corroborate the functional relevance of these states. An early example was the demonstration of ~10 nm steps of myosin along actin seen in the optical tweezers studies of Finer et al. [57]. This finding corroborated ideas for the main force-, and motion-generating conformational change in myosin inferred from X-ray crystallography just a year earlier [74,75]. Together, these studies contributed strongly to establish the, yet today, prevailing swinging lever arm model for operation of the myosin motor. Soon thereafter, ultrastructural studies [76,77] revealed structural states that indicate sub-steps in the motion generation by slow myosins, such as myosin II from smooth muscle and myosin I from the intestinal brush border. The functional relevance of the sub-step was soon corroborated in optical tweezers-based studies [59,60,61] for slow myosin of three different classes (myosin I, smooth muscle myosin II, myosin V). Later, a similar sub-step was observed in faster striated muscle myosins [63,78] using optical tweezers of improved technical performance, something that not yet has been possible to confirm by structural studies due to poor stability of the state. However, there are other pieces of independent evidence for the states associated with sub-steps in fast myosin II [79]. Additionally, the existence of yet other states of fast myosin II in its interaction with actin, has been suggested by single-molecule mechanics [64]. These states do not have counterparts in conventional kinetic schemes based on solution biochemistry and muscle mechanics. However, they are inferred by recent X-ray crystallography-based models for the phosphate-release process and its connection to the force-generating structural change in several myosin classes [39,80]. The above examples demonstrate an important interplay between single-molecule force, and displacement measurements and ultrastructural studies where the former clarify the functional significance of metastable structural states demonstrated by the latter.

Greater level of detail may also be obtained in single-molecule mechanics, e.g., free energy profiles of states and strain-dependent inter-state transitions. Thus, either length ramps [81], sinusoidal length changes [65] or force-clamps with very high time resolution [64], imposed on single molecules of myosin interacting with actin, allow unique quantitative probing of the force dependence of chemical rate constants and free-energy profiles. Also information about the distance between neighboring binding sites for myosin along actin [62,63] and the detailed stress-strain relationship of the actomyosin link [67] can be deciphered.

To conclude this section, a key result of single-molecule mechanics studies is establishment of the functional significance of states and transitions that are inferred from ultrastructural studies. These results would have been difficult to obtain using muscle mechanics or myofibril mechanics. More detailed quantitative information can also be obtained as considered in detail below.

## 3. Challenges in Single-Molecule Mechanics

### 3.1. Directionality of Motor Induced Force and Displacement

A possible complication in single-molecule studies is that the myosin motors are adsorbed to the surface with orientations that are random in relation to the actin filament. This is in stark contrast to the situation in the myofilament lattice with highly ordered arrangement of actin and myosin relative to each other. The importance of this orientation has been illustrated by several experiments. Thus, it was found that actin filaments moved towards the center of native molluscan thick filaments at ~10 times higher speed than in opposite direction i.e., away from the center [82,83]. This finding was soon corroborated using rabbit myosin co-polymerized with molluscan myosin rod into long myosin-rod co-filaments [84]. Additionally, displacements produced by single one-headed or two-headed mammalian myosin, sparsely incorporated into a myosin-rod co-filament, were measured using optical tweezers as function of the orientation of the myosin filament and the actin filament relative to each other [85]. Important findings were that, at close to 0° (the physiological orientation) and 180° motor induced displacement of 10 nm was observed towards the myosin co-filament center. The amplitude of the displacement decreased when the orientation was changed towards 90° in which case 0 nm displacement was observed. The 10 nm displacement observed with properly oriented myosin and actin was twice that of 4–6 nm observed in studies using randomly oriented myosin fragments [58,86,87]. However, interestingly, the latter values are similar to 5 nm averaged over all measured angels (0–180°) in [85].

The significance of the orientation of the myosin motor relative to the actin filament for experimental results from single-molecule mechanics lends support from other studies. For instance, the maximal force that a single actin filament experienced when sliding in the forward direction i.e., toward the center of the thick filament was ~10 times larger than the force in the opposite direction, independent of the number of participating myosin heads [88]. This shows that also force development strongly depends on the orientation of the myosin motor relative to the actin filament. Considerably shorter lifetimes of actomyosin interactions under step loads were found when loads were applied perpendicular to the actin axis [89] in contrast to the case were loads were applied in more physiological relevant geometry i.e., along the actin filament [90]. Other differences between the study (e.g., different surfaces used for myosin attachment and rate of load application) can, however, also contribute to the complexities [89].

It is important in the present context to emphasize that torsional stiffness of a single HMM molecule is extremely small: thermal fluctuations alone can twist myosin 2.8 times [90] making it possible for myosin and/or HMM to interact with actin under a range of orientations e.g., in single-molecule studies. Therefore, unless the orientation of the myosin molecule is constrained by incorporation into a thick filament, the optical tweezers data are likely to reflect averaging over a range of actin-myosin interaction angles.

### 3.2. Surface Immobilization and Surface-Motor Interactions

In addition to the orientation between actin and myosin considered above, the perspective from which the myosin head will “see” actin also depends on its mode of surface immobilization, whether to a glass coverslip or a bead. This effect necessarily varies with the type of motor fragment used. Accordingly, the velocity of actin filaments propelled by short or long S1 molecules bound directly to a nitrocellulose surface is several-fold smaller than that produced by HMM. However, the velocity produced by HMM is ~2-fold higher than that produced by full length myosin molecules [91]. Most likely the latter effect is attributed to interactions between the myosin tail (light meromyosin; LMM) and the actin filament rather than to different modes of interaction of HMM and myosin with the surface [92]. With regard to HMM driven motility on nitrocellulose, evidence was further presented that the motility is supported largely by those molecules that are bound to the substrate near the C terminus (the HMM-LMM junction) [93]. The importance of the mode of surface protein interaction is further emphasized by the effect of S1 binding to the surface via the biotin-avidin system linked to the regulatory light chain rather than via non-specific adsorption [94]. This specific linking of S1 via the regulatory light chain gave the same actin filament velocity as produced by full length myosin but appreciably faster velocity than produced by non-specifically adsorbed S1. Not surprisingly, the above cited results suggest that non-specifically adsorbed S1 and HMM are bound to nitrocellulose films in several configurations and support motility to different degrees. This is naturally also the case for non-specifically adsorbed myosin fragments in single-molecule mechanics studies. The phenomenon has been nicely demonstrated [93] using proteolytic digestion of HMM and S1 adsorbed to nitrocellulose, followed by sodium dodecyl sulfate-polyacrylamide gel electrophoresis (SDS-PAGE) to identify the digestion products. Consistent with the above results, the nature of the motor adsorbing surface also has critical effects on the actin propelling function of different myosin fragments. For instance, HMM-propelled actin filament sliding speed increased with the surface hydrophobicity as measured by the water contact angle of the surfaces (in the range of 20–80°) [95]. It was suggested that the higher contact angle surfaces (e.g., trimethylchlorosilane-[TMCS-] derivatized hydrophobic surfaces) promote HMM attachment via its C terminal (HMM^C^) while on low contact angle surface (e.g., hydrophilic negatively charged surfaces—SiO_2_) the HMM^C^ would be supplemented with electrostatically adsorbed HMM molecules via the N terminal (HMM^N^) with no actin binding and thus lower sliding velocity [95]. HMM adsorption to TMCS mainly via its C-terminal was supported in a more recent study [96] also showing that HMM on such surfaces hold actin filaments ~40 nm above the TMCS-surface with the catalytic site being 20–30 nm above the surface. Finally, the surface adsorption mechanism of myosin constructs affects their catalytic activity. A majority (80%) of adsorbed HMM molecules on a TMCS functionalized surface show similar or slightly higher average steady-state turnover rate than in solution. However two other much slower rates were observed supporting the idea of different HMM configurations on the surface [97]. This was further emphasized by increased amplitude of the latter phases on a negatively charged SiO_2_ surface compared to the TMCS surface [96]. The choice of surface in single-molecule optical tweezers studies is thus important. In addition to surface hydrophobicity also surface charge [98,99] and nanomechanical properties of the surface may be of relevance [100,101,102].

Bead displacements in optical tweezers set-ups may underestimate the myosin induced displacements due to the system compliance, e.g., because the linkages between the actin filament and the bead [103] and between the myosin molecule and the surface are not rigid [85,104]. Application of tension to the filament [57,103,105] counteracts these problems. However, it may also cause microspheres to rotate in the trap with filament bending near their attachments to accommodate this rotation and possible effects on event detection [103]. Recently, streptavidin-coated quantum dots were attached to biotinylated actin filaments enabling observation of the actual filament translation. This, in turn, enabled that the effect of compliance due to bead-actin linkage could be eliminated in the analysis [67]. By this effective workaround detailed characterization was possible both of the myosin working stroke and the elastic characteristic of the actomyosin cross-bridge (see further below).

From the above, step lengths and forces produced by single myosin molecules or motor fragments vary between studies, not only due to different detection and quantification methods but also due to motor constructs and geometries used as well as the mode of surface immobilization and/or the surface properties. Observed variabilities are tabulated in detail in [106] and briefly considered below in comparison to estimates from muscle mechanics studies.

### 3.3. Solution Compositions Differing from that In Vivo

In cells, myosin and actin work in a dense macromolecular environment. Observed effects of crowding and confinement on molecular reaction rates and equilibria have gained increasing attention [107]. Molecular crowding causes increase in the maximum velocity of the actomyosin ATPase cycle as the myosin head acquires a more compact conformation in the crowded environment than in a dilute solution [108]. However, the effects of crowding have not, to the best of our knowledge, been considered in single-molecule mechanics. Other important factors in addition to crowding are the ionic strength and temperature [109,110,111] that affect several aspects of actomyosin kinetics. Most single-molecule mechanics studies have been performed at ionic strengths (usually below 50 mM) and temperatures (usually 20–25 °C) below physiological values (reviewed in [106]). Again, to the best of our knowledge, no detailed studies have been performed of how these parameters affect single-molecule mechanics. One may instead consider related studies using in vitro motility assays. Some data from such studies suggest that velocity increases with increasing ionic strength from 10 mM to ~100 mM where it approaches a maximal value [112]. However, other studies found minimal changes in sliding velocity in the range of ionic strength between 40 and 130 mM [113] yet other studies suggest a substantial increase in velocity with increased ionic strength in the range 20–100 mM [79] or 40–80 mM [92] and reduced velocity at higher ionic strengths.

Filament velocity in the vitro motility assay increases with increasing temperature [112] but the temperature dependence of sliding velocity differs for slow and fast isoforms [114]. The temperature dependence of velocity using pure actin filaments (without regulatory proteins) deviated from linearity in the Arrhenius plot with a breakpoint at 25 °C [114,115]. Notably, this is close to temperatures often used in single-molecule mechanics [106]. Because similar effects have been observed for the unloaded shortening velocity in muscle [116], it is not necessary to invoke temperature dependence of surface adsorption mechanisms of the myosin motor fragments [96] to account for these findings. However, linear Arrhenius plots were obtained using regulated thin filaments in the in vitro motility assay [117], indicating roles of troponin and tropomyosin in modulating the actin-myosin interaction by mechanisms different from simple on/off switching. It is pertinent in this connection to mention that most single-molecule mechanics studies have been performed using actin filaments without regulatory proteins (however, see [118]; more details below).

### 3.4. More on Lack of Accessory Proteins in Single-Molecule Mechanics

As indicated above, accessory proteins such as troponin and tropomyosin are likely to have modulating roles in muscle contraction different from on/off switching of actomyosin interactions upon changes in intracellular Ca^2+^—concentration. There are also other proteins that are normally not present in mechanical studies of isolated proteins, but that have potentially important modulating roles in contraction. Such proteins include titin and MyBPC. Both may play roles [119,120,121] in controlling the degree to which myosin heads leave their sequestered (parked) super-relaxed state [40,41] on the thick filaments under different conditions, e.g., in case of stress or need for increased power. These properties are of increasing interest to study using in vitro systems with isolated proteins because of potentially important roles in effects of myosin active drugs [21,24,40,122] and/or in cardiomyopathies [30,31,38,123,124,125,126,127]. In these regards the degree of phosphorylation of the regulatory light chains of striated muscle myosin is also of interest to control as this property affects both the state of activation of the thick filaments and drug effects [38,128,129,130,131,132,133]. In addition to the possibility of controlling phosphorylation status and adding MyBPC or titin fragments in single-molecule mechanics studies a possibility may be to use isolated native thick filaments containing several of the components [5].

### 3.5. Time Resolution

Thermal and instrumental noise limit the temporal resolution of optical tweezers in force and displacement measurements. This is a particular challenge when observing non-processive motors such as muscle myosin II, which is characterized by short interaction lifetimes. Thus, each interaction between actin and myosin lasts less than 20 ms on average under physiological conditions, and the displacement of the actin filament is even faster [64,78,134,135]. The working stroke of muscle myosin is associated with the formation of molecular bonds with actin and follows binding on the sub-millisecond timescale. The classical approach for the detection of interactions of muscle myosin and actin filaments based on reduced thermal noise [58] is slow in comparison. Thus, position variance is calculated using time windows Δt > 5 ms, and events with shorter durations cannot be detected [72,136]. To study effect of load on actomyosin biochemical transitions load-clamp may be applied several milliseconds after detection of the molecular-bond formation adding further time delay [61]. Thus, until recently the experiments were usually carried out at low [MgATP] effectively probing only slow transitions. Recently, however, new innovative approaches have been developed. Greenberg et al. [78] calculated the parameters at saturating 4 mM MgATP based on maximum likelihood estimation (MLE) fitting that should yield correct values despite limitations of the experimental temporal resolution (10–20 ms). As an alternative and more direct approach, an ultrafast force-clamp spectroscopy instrument was built to measure load dependence of the myosin working stroke [64]. The measurement delay of only ~10 μs allows detection of very short interactions (~100 μs) together with ability to detect sub-nanometer conformational changes. Constant loads can be applied to a single motor domain of myosin before its working stroke is initiated, thus directly measuring working stroke load dependence. Depending on the applied load, myosin weakly interacted (<1 ms) with actin (i) without production of movement; (ii) fully developed its working stroke or (iii) prematurely detached. 

A technically simpler approach (without feedback control) than that of Capitanio et al. [64] was developed in [65]. With its temporal resolution of 5 ms this method, denoted “harmonic force spectroscopy”, has sufficient temporal resolution to detect the load-dependent adenosine diphosphate (ADP) release rate of human β-cardiac myosin II (being slower than fast skeletal muscle myosin II). The simplicity of the method may prove useful for examining differences in strain-dependent detachment kinetics of wild-type and diseased myosins carrying cardiomyopathy mutations.

## 4. Mechanical Experiments on Muscle Cells and Myofibrils—Concepts, Main Findings and Challenges

The experiments using single molecules are important for deciphering the elementary mechanisms in the myosin-actin interactions. However, as pointed out in the introduction, myosin and actin in the muscle cell work in an environment with many other proteins and a highly organized structure. One may therefore argue, that in order to understand the myosin-actin interaction and muscle contraction, it is important to investigate their mechanisms in cellular and sub-cellular preparations. In this context, single muscle fibers (cells), isolated myofibrils and sarcomeres—the smallest muscle structures that still maintain the three-dimensional lattice and most proteins intact—have all been used for mechanical experiments to elucidate how the molecular mechanisms of contraction scale up to ordered structures (e.g., [137,138,139,140]). While several of these studies focus on muscle adaptations to different conditions (e.g., disease, temperature, fatigue, etc.), some have focused on myosin-actin interactions and the effects of strain on force production, which is highly relevant for the understanding of molecular mechanisms of contraction.

**Figure 4:**
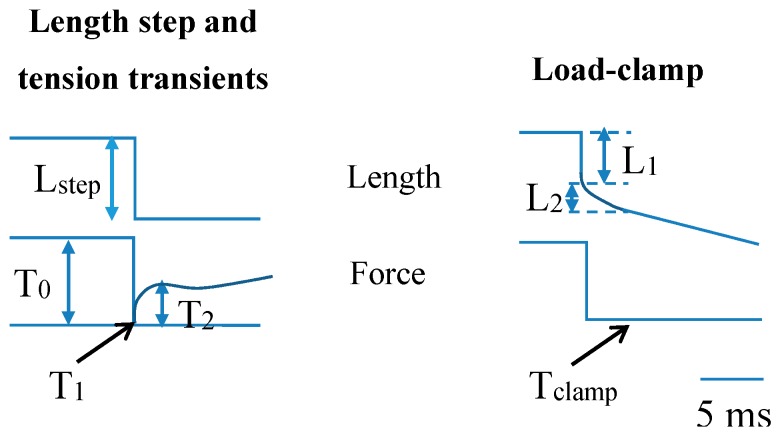
Schematic illustration of length step experiment (**left**) giving rise to tension (force) transients and load-clamp experiment (**right**) giving rise to velocity transients of muscle cells followed by steady-state shortening at constant velocity. In the length-step experiment length is controlled and the resulting tension transient includes a drop of tension from the isometric value, T_0_ down to the extreme tension level at the end of the length step, T_1_. This is followed by a subsequent rapid recovery to the intermediate tension level T_2_ (see further text). In the load-clamp experiment, load (force) is controlled, and the resulting length changes are recorded, e.g., the initial elastic response (amplitude L_1_) when force drops and the subsequent rapid phase of shortening (phase 2; ending at length L_2_) before slower shortening at a steady-state rate.

**Figure 5:**
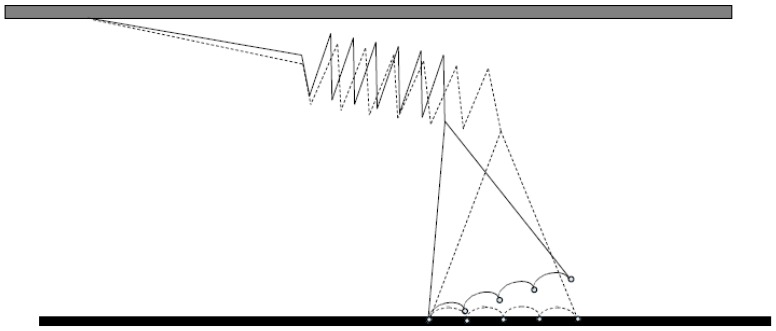
Force generation by myosin on actin as tentatively depicted in [141]. A larger part of the myosin head swings while stretching an elastic element in series and, step-wise, increasing its strength of binding to actin.

In early experiments, intact amphibian muscles [141,142,143,144,145] were primarily used. Single muscle fibers were mechanically dissected from these muscles and then electrically excited as the membrane was intact. After full activation by high-frequency electrical stimulation, different mechanical perturbations were imposed on the preparations. In a set of classical experiments, Huxley and Simmons [141] and later Ford et al. [142,146,147] imposed rapid length changes and recorded the resulting tension transients (Figure 4, left) under different conditions, thus deriving information about cross-bridge operation. Upon rapid shortening the force changes in four phases: (1) during the fast shortening step, there is a force drop whose magnitude increases with the shortening amplitude; (2) during the next 3–5 ms there is a rapid force recovery; (3) during the next 10–50 ms there is an extreme reduction of force recovery; and (4) during the remainder of the response, there is an asymptotic recovery towards maximum isometric force. At the end of phase 1, a maximal drop in force (T_1_) is observed and the beginning of phase 2 indicates a transition into recovery of force back towards the tension level before the length change. A following inflection or even a low peak in the force time course at force (T_2_) indicates the transition into phase 3. These observations have been central in the development of modern cross-bridge models of contraction [141,142] (see further below), and suggested that the mechanism responsible for force production during myosin-actin interaction is strain-dependent and occurs in several steps (Figure 5). If load is suddenly changed instead of length, a related transient, the so-called velocity transient, is obtained (Figure 4, right). This is considered further below in relation to models and in relation to efforts to measure the cross-bridge power-stroke distance by either single-molecule mechanics or muscle mechanical measurements. Other sets of classical experiments include studies of the relationship between the force developed by the muscle and the shortening or lengthening velocity (the force-velocity relationship) related to the energetics of contraction and ultrastructure [143,145,148,149,150,151,152,153,154,155,156,157,158,159,160]. Such experiments have been of critical importance for development of models for muscle contraction [156,161,162,163,164,165,166,167,168,169]. By being an ensemble property, the force-velocity relationship has no direct counterpart in single-molecule experiments. However, in this connection it is worth mentioning single-molecule experiments that elucidate the strain-dependence of rate constants for cross-bridge detachment, as these kinetic properties are of critical importance in determining the force-velocity relationship [64,65]. Also recent experiments using small groups of isolated myosin motors are of interest as they as they constitute an experimental system intermediate between single molecules and the large actin-myosin ensembles of muscle [170,171]. Presently, length ramps performed at constant velocities on muscle cells or myofibrils are commonly used for studying the molecular mechanisms of muscle contraction [172,173,174]. These length ramps lead to force responses that are qualitatively related to early studies that used step shortening (tension transients in Figure 4), with force decreasing in proportion to shortening, in the first phase and a slow down until an asymptotic approach to a lowered, but constant steady state. Some of the studies applying length ramps show a transition in the force trace from phase 1 to phase 2 (hereafter called critical point P_1_) that occurs at a critical sarcomere length, and a transition in the force trace from phase 2 to phase 3 (hereafter called critical point P_2_), that occurs at a second critical sarcomere length [174,175]. While phase 1 in force traces is commonly associated with a purely elastic response, the behavior during phase 2 is attributed to a repartitioning of cross-bridges from the pre to the post-power stroke state, due to an acceleration of the power-stroke step under conditions of lowered mechanical load on myosin cycling.

These studies are on agreement with the general observations described earlier in this paper in studies looking into individual myosin-actin interactions. In one study [175], the mechanisms responsible for the force transients during a shortening ramp was investigated after fibers were treated with the myosin inhibitor blebbistatin, which biases cross-bridges into a pre-power-stroke state. The authors observed that the P_1_ transition was significantly decreased during shortening, suggesting that it is associated with the cross-bridges populating the different stages during the cross-bridge cycle.

When muscle fibers or individual myofibrils are stretched during full activation at low speeds (~2 length (*L*o)/s at 5 °C), cross bridges have to resist the opposing forces while attached to actin. Under these conditions, force increases in two phases: (1) a fast increase that happens over the extension of a few nanometers and then (2) a slow increase or a stabilization of force [175,176,177,178,179]. The transition between the two phases is marked by a change in slopes of the force rise and is commonly associated with the mechanical detachment of cross bridges from actin. The detachment happens after the cross bridges reach a critical extension length (*L*c), commonly observed in lengths between 8 and 20 nm/half-sarcomere (HS), depending on the experimental condition [176,178]. These observations also point towards a mechanism in which the cross-bridge cycle is load-dependent, with strain having a direct effect on the myosin-actin interaction and force production. The resistance to stretch of active muscle is of great fundamental importance. It occurs as part of almost all smooth motions powered by skeletal muscle and is of dominant importance in e.g., downhill running and postural reflexes. However, as further elaborated on below, the mechanisms are poorly understood, and several hypotheses have been and are under consideration.

One issue that complicates muscle mechanics results and that is not captured by single-molecule mechanics is the effect of non-uniform sarcomere properties along a muscle cell. The feature may for instance be of importance during stretch of active muscle [180,181,182], during tension relaxation after drop in calcium concentration [144] or following increase in the concentration of inorganic phosphate [183,184] in myofibrils and skinned muscle cells. Furthermore, the possibility exists that sarcomere non-uniformities or differences between cells in series in the cardiac ventricle contribute to pathogenesis of disease [185,186,187,188,189,190,191] possibly worsened by mechanical instabilities [35] with more than one stable velocity for a given load.

Challenges in the interpretation of muscle mechanical experiments in terms of actomyosin cross-bridge function, such as the need to average over a wide range of cross-bridge states and strains, are considered below.

## 5. Key Cross-Bridge Characteristics from Single Molecules to Muscle

### 5.1. General

A cross-bridge develops its highest force under isometric conditions, i.e., without sliding of actin and myosin relative to each other. The force-generation is believed [141] to result from step-wise straining of an internal elastic element in the cross-bridge (Figure 5). On this assumption, force can be expressed as the stiffness of the strained elastic element (“the cross-bridge stiffness”) times the strain of this element.

Estimates of cross-bridge stiffness and maximum force per cross-bridge have been obtained using both muscle cells and single molecules (reviewed in [192]). A large fraction of the muscle fiber experiments has been performed using frog muscle [192,193] whereas all single-molecule studies of muscle myosin have used isolated proteins from mammalian muscle. Because there may be differences in mechanical properties between frog and mammalian muscles [192] we primarily consider measurements on mammalian muscle below. We also focus on studies from 1994 and onwards due to findings around that time [194,195,196] that the myofilaments are appreciably more compliant than previously believed [197] contributing 50–70% of the total sarcomere compliance in isometric contraction [198,199] leaving only 30–50% to the cross-bridges.

### 5.2. Cross-Bridge Stiffness

The estimates of cross-bridge stiffness based on single-molecule mechanics vary between 0.2 and 2.9 pN/nm [57,58,63,67,105,200] (reviewed in [106]) with most values near the lower limit referring to single myosin heads (S1; although see [200]; 1.7 pN/nm). The values 1.3 pN/nm [105] and 2.9 pN/nm per head [67] have been reported for single molecules of full length myosin. Among the single-molecule results, low values are generally attributed to compliant elements in series with the actin-myosin system (attachment of proteins to substrates, e.g., beads) whereas the highest value (2.9 pN/nm) [67] was obtained in experiments designed to minimize the effects of such compliant elements. Interestingly, the cross-bridge stiffness suggested by that study is higher than the cross-bridge stiffness estimated using fast rabbit muscle fibers (1.7 pN/nm) [199], although lower than the values for cross-bridge stiffness deduced from experiments on frog muscle cells (3.3–5.0 pN/nm) [155,173]. Lower cross-bridge stiffness values have been found for slow muscle myosin both in single-molecule studies [63] and in muscle mechanical experiments [201], an issue that deserves further investigation (see [192]). In the following we focus on fast muscle

The highest cross-bridge stiffness values have been obtained in the absence of ATP (rigor conditions). Although Kaya and Higuchi [67] did not detect any appreciable change in cross-bridge stiffness by adding 1 mM ADP (reduction from 2.9 to 2.6 pN/nm), one cannot exclude the possibility that stiffness is different in e.g., actomyosin-ADP-Pi states or in actomyosin-ADP states (cf. [79,202]) formed during active contraction. Another complication for muscle experiments is that the actual number of attached cross-bridges that contribute to stiffness in muscle fibers in rigor is not known with 100% certainty [203,204,205] despite evidence suggesting that all myosin heads bind to actin in rigor [206]. For instance, the geometrical arrangement of the myofilaments in the sarcomere may lead to binding of the two heads of one myosin molecule either to different actin filaments [207] or to one given actin filament [208]. In both cases, such binding may lead to structural changes in the lever arm region of the myosin heads, with possible changes in stiffness per head. However, a recent study using fast rabbit muscle fibers [199] lend support to the view that the stiffness of two-headed attachment is twice that of one-headed attachment. Therefore, we assume that cross-bridge stiffness in rabbit muscle is about 2 pN/nm (per head) (see also [192]). However, in view of the seemingly well controlled measurements by Kaya and Higuchi [67] on single full length myosin molecules the cross-bridge stiffness may actually be as high as 3 pN/nm.

### 5.3. A Simple Model

**Figure 6:**
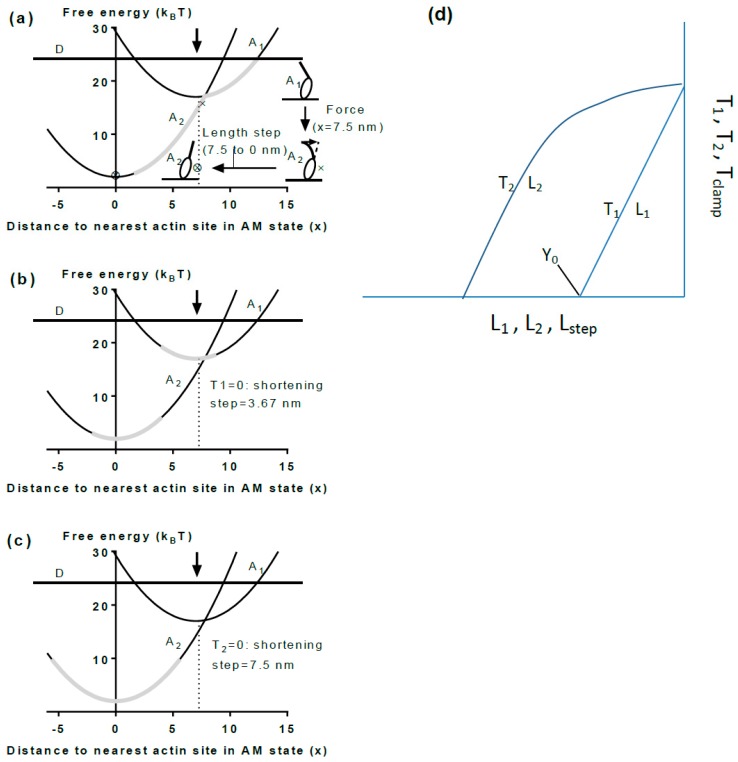
Simplified tentative cross-bridge model and details of muscle mechanical experiments for analyzing model. (**a**) Free energy diagrams for one detached cross-bridge state (straight horizontal line at 25 k_B_T; state D) and two attached cross-bridge states, a pre-power-stroke state A_1_ and a post-power-stroke state A_2_. The free energy values are given as function of the variable x, reflecting the distance of the myosin head from the nearest binding site on actin, Definition of x = 0: free energy minimum and zero force in state A_2_. Arrow indicates mean position for attachment into state A_1_ from the detached state. Grey areas on free energy diagrams indicate range over which different states may be populated in a muscle during isometric contraction. Inset: Schematic structural changes associated with length step (power-stroke) and isometric tension development (tensing step); (**b**) Same diagram as in (**a**) but indicating cross-bridge distribution in muscle (grey area) after a shortening step that brings tension to zero; (**c**) Same diagram as in (**a**,**b**) but displaying the cross-bridge distribution after rapid recovery subsequent to length step (mentioned in (**b**)) or after rapid shortening during phase 2 of a load-clamp from isometric force to zero force; (**d**) Schematic plots of T_1_ and T_2_ (see Figure 4) against the imposed length changes. Note similar relationships between T_1_ and T_2_ and the imposed length steps (L_step_) and between the imposed loads (T_clamp_) in the load clamps and the recorded length changes L_1_ and L_2_ (cf. [173]).

Parameter values such as cross-bridge stiffness, the power-stroke distance and force per cross-bridge can be usefully related to a simple structural and mechanochemical model assuming two actin-attached states of myosin. A model of this type, based on original ideas in [141], is described in Figure 6a with a pre-power-stroke state A_1_ and a post-power-stroke state A_2_. In this model, we do not consider the entire cross-bridge cycle but only the events from cross-bridge attachment to force-generation in a simple three-state scheme. Most likely there are more states between the detached state and the A_1_ state [39,80,209,210] but these are not considered here. Free energy profiles (G_i_(x)) for the attached states i = 1,2 are indicated in Figure 6: G_i_(x) = G_i_(x_i_) + (k/2)(x − x_i_)^2^ where k = 2 pN/nm is the cross-bridge stiffness (see above), G_1_(x_1_) = 17 k_B_T and G_2_(x_2_) = 2 k_B_T are the free energy minima for the two states, consistent with the free energy (25 k_B_T) available from the turnover of 1 ATP molecule under physiological conditions. Finally, x_1_ = 7.5 nm and x_2_ = 0 nm are the distances of the free energy minima from the nearest actin-binding site (defined as distances from the position where force in state A_2_ is 0 pN). Cross-bridge force is given by the derivative, i.e., the slope, of the free energy diagram at the relevant x-value. As will be shown below, the distance x_1_ − x_2_ = 7.5 nm approximates the power-stroke or working stroke distance in such a model. 

### 5.4. The Model and Single Molecules

**Figure 7:**
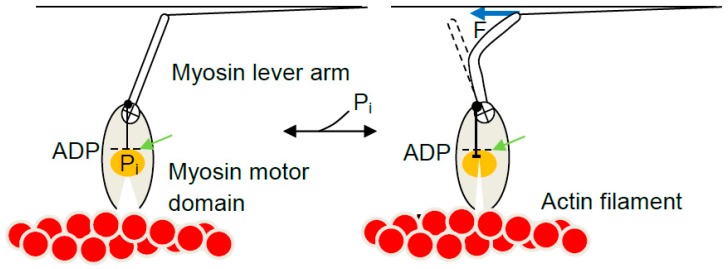
Tentative force generating mechanism of the Eyring type. A small structural change in the motor domain close to the ATPase site (green arrow) is amplified (by rotation of circle with cross) to strain an elastic element (tentatively by bending the lever arm). In the absence of resisting tension the entire lever arm relaxes into the post-power-stroke orientation. Pi: inorganic phosphate.

In terms of the model in Figure 6, each myosin head in single-molecule studies attaches to actin in the state A_1_ at a given x-value. Because the attachment step is most likely rate limiting for the cycle [210], the subsequent inter-state transitions would be comparatively rapid [165,211] consistent with observations in ultra-fast force spectroscopy of single molecules [64]. For instance, if the free energy of the state A_2_ is lower than that of state A_1_ at the x-value where the cross-bridge attaches, a virtually immediate transition from state A_1_ to state A_2_ will follow. It is of interest to consider two special cases. First, if no load counteracts sliding of actin and myosin relative to each other (e.g., with a very compliant optical trap) the change of state will be associated with an immediate change in x-position to x = 0 nm, to make the force equal to the zero counteracting load. Cross-bridge attachment is usually believed to be most probable at an x-value (here x = 7.5 nm) corresponding to the position for the free energy minimum of state A_1_ ([162,166]; arrow in Figure 6a). If the myosin head attaches into state A_1_ at this x-value, a transition to A_2_, will be associated with an immediate length step of 7.5 nm if there is no load resisting the displacement. This is the average power-stroke distance (x_1_ − x_2_) that can be directly estimated in optical tweezers studies at very low trap stiffness. The observed length steps are expected to vary around this average value because attachment into the state A_1_ occurs over a range of x-values. For attachments at x < 7.5 nm the observed step would be shorter than 7.5 nm whereas for attachments greater than 7.5 nm the step would be longer. Thus, unless detaching (by reversal of the attachment transition), a cross-bridge in state A_1_ at x > 7.5 nm would first cause actin filament movement to reduce x to 7.5 nm followed by rapid transition into the A_2_ state and subsequent sliding to x = 0 nm. The second special case of particular interest is isometric conditions. Under these conditions, a change in state between A_1_ and A_2_ is not associated with any sliding between actin and myosin due to a counteracting load. In optical tweezers studies, this condition is achieved using very stiff traps and load clamp designs that prevent movement of the trapped beads and disturbances, upon changes in force, due to compliant attachments between the proteins and the substrate (cf. [105]). The maximum cross-bridge force upon change in state from A_1_ to A_2_ under isometric conditions (without change in x) corresponds to the change in slope between the curves G_1_(x) and G_2_(x) at that value of x. In structural terms, this transition, denoted a tensing step in [192], is most easily thought of as a sudden structural change in the motor domain (presumably initiated by Pi-release) [80] that, by change of the preferred equilibrium position of the lever arm, causes a sudden straining of the elastic element (e.g., bending of the lever arm; Figure 7). Notably, this course of events is consistent with an Eyring rate model [162,164,212] but differs from that of a Kramers rate model (cf. [213]). In the Eyring model [213] a local chemical change (e.g., release of inorganic phosphate from the active site of myosin) produces a strained state followed by slower relaxation of a larger part of the protein structure into a new equilibrium conformation. In contrast, in a Kramers process [213] a large-scale (several nm) diffusion (e.g., rotation) of a nanoscale domain into the strained state [141,156,169] precedes the local chemical change. The Kramers view was taken in [141] where the chemical change was assumed to follow rotation of a substantial part of the myosin head with straining of an elastic element in series (Figure 5) [141]. Therefore, also isometric force generation by the myosin head is sometimes denoted as execution of a “power-stroke” although this may not be fully appropriate [192] in relation to the current structural perception of the process. The highest possible cross-bridge force for the model in Figure 6 would be expected for cross-bridges attaching at x ≈ 7.5 nm where force immediately after attachment would increase from zero in state A_1_ (slope of G_1_(x) at x = 7.5 nm) to 15 pN (2 pN/nm × 7.5 pN/nm) in state A_2_ (slope of G_2_(x) at x = 7.5 nm). For x < 7.5 nm, the force developed in the tensing step is self-evidently less than the value at x = 7.5 nm. Importantly, lower force than 15 pN would also be generally expected for attachment at higher x because the cross-bridge will not be able to make the transition into the A_2_ state because of its appreciably higher free energy than that of the A_1_ state at x > 7.5 nm.

Estimates of the average isometric force developed by a myosin head of full length myosin vary between 3 and 9 pN in single-molecule studies (cf. [106]) probably because the measured value is affected to different degrees by compliant element in series with the cross-bridge. This includes effects of low optical trap stiffness and/or compliant substrate attachments for both myosin and the actin filament. The highest value (9 pN) was derived in a study [105] that took pain-staking efforts to reduce the mentioned complications. Importantly, the mentioned values are average values whereas individual isometric force events up to 17 pN were observed [105] in reasonable agreement with a power-stroke distance of 7.5 nm and a cross-bridge stiffness of 2 pN/nm (corresponding to force level of 15 pN), as in the model in Figure 6. The average isometric force and the cross-bridge stiffness are related to each other via the average cross-bridge strain. Assuming that the cross-bridge stiffness is ~2 pN/nm and the average isometric force is that observed in the single-molecule studies at room temperature (9 pN), the average cross-bridge strain for an ensemble of cross-bridges would be 4.5 nm. To summarize, we emerge with an estimate of the average cross-bridge force in the range 7–9 pN, most likely close to 9 pN and a maximum force of up to 17 pN, consistent with a cross-bridge stiffness of 2 pN/nm and a power-stroke distance of about 8 nm. The latter value is consistent with the range 6–10 nm calculated by Kaya and Higuchi [106] on the basis of ultrastructural data. It is also consistent with ~9 nm obtained by scaling down (cf. [214]) the more easily measured power-stroke distance in myosin V (25 nm) [60,215] to the shorter length of the lever arm in myosin II (~9 nm [106] compared to 24 nm [215] in myosin V). The power-stroke distance includes a second small step (5 nm in myosin V) [60] gating ADP release.

### 5.5. The Model and Muscle Mechanics

The average isometric force per attached cross-bridge has also been estimated in muscle cells. In such analysis the number of attached cross-bridges has generally been estimated from the muscle fiber stiffness by comparing to rigor stiffness (cf. [199]). Implicit in this analysis is that all myosin heads contribute to stiffness in rigor and that the cross-bridge stiffness under those conditions is identical to that during active contraction. Finally, the total number of myosin heads per half-sarcomere over the fiber cross-section needs to be estimated (e.g., from myofibril density, overlap, etc. (cf. review of such analysis in [192])). Under these (and some further) assumptions, an average isometric cross-bridge force of 6.6 pN has been estimated [199] in fast rabbit muscle at 19 °C, similar to the value of about 6 pN in frog muscle fibers at 4 °C [155]. The fact that these values are lower than those suggested by single-molecule studies (9 pN; [105]) could be due to overestimation of the number of attached cross-bridges during isometric contraction in muscle. There may also be reduced sampling of cross-bridges in low force states in the single-molecule studies due to lower affinity in these states compared to the situation in the myofilament lattice of muscle. One could also use an approach based on energetics [216] to estimate isometric force per cross-bridge and the power-stroke distance. The thermodynamic efficiency lies in the range 40–60% [217,218] for fast mammalian muscle and the free energy of ATP turnover is ~25 k_B_T. Thus, 25 × 0.4 k_B_T < k d^2^/2 < 25 × 0.6 k_B_T, where k is cross-bridge stiffness, d is the power-stroke distance and 1 k_B_T ≈ 4 pNnm. Assuming that cross-bridge stiffness is 2 pN/nm, it follows that 6.3 nm < d < 7.8 nm which is consistent with the estimates of 7–8 nm from single-molecule mechanics using full length mammalian myosin [67,105].

The range of cross-bridge strains existing simultaneously during isometric contraction in a muscle cell is related to uniform distribution of the myosin-binding sites on actin relative to the nearest detached myosin head. Such uniformity is caused by mismatch of the periodicities of the thin and thick filaments and factors related to lack of complete register between myofilaments and sarcomeres over the muscle cross-section ([166,219], Supporting Information, [164]). In experiments using muscle cells, the average cross-bridge strain may be estimated from the amplitude (Y_0_) of a rapid shortening step for which the tension level T_1_ (Figure 4 and Figure 6d) is zero. If there are no inter-state transitions during the length change, approximately true for very fast length changes (duration < 0.1 ms), the length change of the cross-bridge elasticity (from distribution in Figure 6a to that in Figure 6b) would correspond to the average cross-bridge strain, Y_xb_, during isometric contraction. To obtain the latter value from muscle experiments, the Y_0_ value must be corrected by subtraction of the strain in the myofilaments. Naturally, such corrections introduce uncertainties, independent of approach used (cf. [67,167,192,198,220,221,222]). However, interestingly the resulting estimate of Y_xb_ of 5.2 nm in skinned rabbit psoas muscle fibers at 19 °C [199] is very similar to 4.5 nm calculated from isometric cross-bridge force and stiffness from single-molecule data (see above).

In accordance with the model in Figure 6 the average isometric cross-bridge strain in muscle cells is lower than the step lengths of 7–10 nm [67,85,105] measured in single molecules using very compliant optical traps. The step lengths reflect the power-stroke or working stroke distance defined in [106] as the “the limit of the displacement generated by the conformational changes of the myosin head“. Thus, under these nearly unloaded conditions, with very compliant traps, the entire structural change in the myosin motor is used for actin filament sliding without straining of the elastic element (cf. inset Figure 6a, dashed arrow). In muscle mechanical experiments the power-stroke distance is reflected in the minimum amplitude of a shortening step for which the tension level, T_2_, at the end of the fast (a few ms) force-recovery after the length step, reaches zero (Figure 4 and Figure 6d). However, the latter value is affected by changes in cross-bridge distributions between different attached and detached states [223]. The working stroke distance can also be obtained in muscle cell experiments during the first phases of the length response of the sarcomeres following a sudden change in the clamped load from the isometric value to very near zero load (Figure 4). First, there is an initial elastic response with sudden drop in tension to near zero corresponding to a change in the cross-bridge distribution from the grey area in Figure 6a to the grey area in Figure 6b. Then there is a phase of rapid shortening (phase 2) at constant load before onset of steady-state shortening at lower speed (Figure 4, right). The phase 2 would correspond to a shift of a dominant part of the cross-bridge distribution from the grey area in Figure 6b to that in Figure 6c. Usually [155,156,173], the amplitude of the rapid shortening during phase 2 is taken as an estimate of the working-stroke distance giving a value of ~8 nm in rabbit psoas muscle [156]. However, it may be inferred from inspection of the free energy diagram in Figure 6 that the definition of the working stroke in the single-molecule studies (x_1_ − x_2_; 7.5 nm in Figure 6) does not directly correspond to the amplitude of phase 2 shortening in load-clamped muscle fibers (difference between Figure 6b,c). Clearly the latter amplitude only corresponds to a fraction of the power-stroke distance x_1_ − x_2_. Appreciably better correspondence would be obtained if the working stroke in muscle cells is taken as the sum of the elastic response and the subsequent shortening during phase 2 of the load clamp (7.5 nm left-ward shift of grey areas on free energy diagrams from Figure 6a–c). Therefore, using the same definition as for single-molecule results, the data in [156] suggest a power-stroke distance of ~12 nm (3.8 nm from the initial elastic response [199] and 8 nm from phase 2 shortening [156]). Thus, if the working stroke distance is defined in similar way in terms of the model in Figure 6 for single molecules and muscle fiber load-clamp data, the latter data give 4 nm higher values than the most recent estimates in single-molecule studies.

Although this discrepancy is rather small, it is highly important when defining cross-bridge models similar to that in Figure 6, and for clarifying whether there is a need to sub-divide the power-stroke distance into several sub-strokes (see further below). One possible explanation for a longer distance in muscle cells could be effects of the ordered myofilament lattice and the presence of accessory proteins. Alternatively the single-molecule studies underestimate the working stroke distance. It should be mentioned in this connection that one study using full length myosin [135] and one study using HMM [57] have given estimates of approximately 11 nm. Furthermore, one study using thin filaments [118] detected a power-stroke distance of 13.7 nm after activation of the thin filaments by other strong-binding cross-bridges at micromolar MgATP. It is likely that a range of methodological differences [106] account for the rather wide range of power-stroke distances observed. Based on the overall evidence reported in this section we lean towards an average power-stroke distance in the approximate range 7 nm [105]–8 nm [67]. These values were observed in studies using full length myosin which took special precautions to eliminate effects of compliant elements and with particular focus on detailed quantitative analysis. The studies reporting larger power-stroke distances had other foci, e.g., pioneering single-molecule studies of actin-myosin [57] or investigating differences between smooth and skeletal muscle myosin [135] or effects of troponin-tropomyosin [118] on actomyosin interactions.

One also needs to consider the validity of the muscle mechanics data suggesting working stroke distances of 12 nm or greater (see above). One possible contributing factor is if the negatively strained cross-bridges detach very rapidly (cf. [223]). This may require extra sliding during phase 2 before a steady state is attained. It is also of interest to compare to frog muscle fibers for which T_2_ curves have been obtained under carefully sarcomere length-controlled conditions. In these experiments zero T_2_ level was seen for a length step of ~10.5 nm [173,224] consistent with a working stroke of less than 10 nm if myofilament elasticity is considered. Interestingly, in one such study [173], the length change (10.5 nm in [173]) that brought the T_2_ tension to zero was approximately equal to the sum of the elastic response and phase 2 shortening during a load clamp to ~zero force. Considering that at least 2 nm of the 10.5 nm length change would correspond to filament compliance (50% of Y_0_ of ~4 nm in the same study) the working stroke would be 8–9 nm in reasonable agreement with single-molecule data for full length myosin [67,105]. One may of course argue that such a short working stroke is a characteristic only of frog muscle whereas mammalian muscles have a working stroke close to 12 nm as suggested by the above mentioned load-clamp data [156]. However, values of ~12 nm have also been observed in frog muscle fibers using the load-clamp approach in combination with X-ray interference data, e.g., 8–13 nm [225] or 12 nm [155]. These values from frog muscle are consistent with the high values obtained in load-clamp data from mammalian muscle whereas the low values from early frog muscle data [173] are more consistent with the estimates of the working stroke distance in single-molecule studies.

On the basis of the above analysis it first seems appropriate to stress the importance of using the same definition for the working stroke distance when comparing data from different types of experiments. It is also of interest to clarify the variability of 8–13 nm in load-clamp data. Using a definition consistent with single-molecule experiments, the above review of the literature supports a working stroke distance close to 8 nm. This value is likely to include a contribution from a 1–2 nm long sub-step that, so far, has been revealed only in single-molecule data using myosin S1 from skeletal muscle [63]. This second sub-step is most likely related to strain-dependent gating of the ADP release [61,79,226].

It is interesting to consider the current numerical estimates of cross-bridge stiffness and the maximum isometric force in relation to models assuming a working stroke subdivided into two or several sub-strokes [156,167,211,227,228]. First, with the present estimates of maximum isometric force and the cross-bridge stiffness it is clear that a model with just one working stroke is fully consistent with experimental data if the maximum displacement upon the stroke is about 8 nm. This would correspond to events with a maximum isometric force of around 8 × 2 pN = 16 pN, close to what was actually observed (17 pN). It would also correspond to an average cross-bridge strain that is considerably lower than the maximum value of 8 nm. Furthermore, the values for the maximum isometric force and the stiffness put important constraints on models assuming more than one working stroke. For instance, if two sub-strokes of about 4 nm are assumed [211] the maximum force expected in single-molecule studies would be around 4 × 2 pN = 8 pN if the cross-bridge stiffness is 2 pN/nm. The average force per cross-bridge would be considerably lower.

### 5.6. Section Summary

From the above, it appears that there is good correspondence between key parameter values obtained using single-molecule mechanics and muscle mechanical experiments. These parameter values are also in good agreement with simple models assuming a power-stroke occurring in one step by an Eyring-like mechanism (see above [162,164,212]). This idea is consistent with recent ultrastructural data suggesting that force-generation, or lever arm swing with filament sliding, is gated by small structural changes in the myosin motor domain [39,80,209,229] following release of inorganic phosphate from the active site of myosin. Furthermore, there is growing evidence that bending of the lever arm or of a pliant region of the lever arm between the converter domain and the essential light chain underlies the cross-bridge elasticity [230] (Appendix by Howard and Spudich) [201,231,232]. However, there is not yet conclusive evidence to exclude a Kramers type mechanism and many recent models seem to rely on this idea [156,169,211,227]. This is one reason it is important to clarify once and for all if force-generation by the actomyosin cross-bridges is of the Kramers or Eyring type. The clarification of this issue is also of significance because the specific model is constraining the thinking not only of theorists but also of experimentalists when interpreting their data. Most likely, the very nice depiction (cf. Figure 5) of the power-stroke as a Kramers process in the pioneering paper of Huxley and Simmons [141] is yet in the mind of many researchers.

Despite the rather good correspondence between parameter values obtained in muscle/myofibril mechanics and single molecules there are remaining issues to clarify. First, the concept of working stroke/power-stroke and step length contain ambiguities between experimental systems. Another concept that contains ambiguities is the duty ratio. Although the concept is useful for first-order distinctions between motors with different properties, e.g., between processive and non-processive myosins, difficulties arise when efforts are made to apply it to more complex issues. The concept was introduced in relation to the emerging in vitro studies in the early 1990s [233,234]. At the time it was generally used to quantitatively characterize a simple model where each myosin motor is believed to attach in a force generating, strongly bound state, undergo the power-stroke and then detach. In this framework the duty ratio is defined as the fraction of the ATP cycle time that the myosin motor stays attached to actin as quantified by the on-time in single-molecule mechanics divided by the ATP cycle time estimated from the maximum actin-activated ATP turnover rate. Ambiguities first arise when load is applied because that will change the on-time giving more than one duty ratio value for a given motor [235]. Even more importantly, the concept is difficult to apply to an ensemble of myosin motors working together such as the myosin motors of muscle or the myosin motors in an in vitro motility assay [212,236,237]. Under these conditions, direct application of duty ratios derived using single-molecule on-times cannot predict the high sliding velocities observed [165,238]. The reason is that in an ensemble of motors, several of them are still propelling the actin filaments when some motors have reached the end of their power-stroke (x = 0 nm in state A_2_ in Figure 6). The latter will therefore be brought into a region of negative strain, executing a so called drag stroke (to be clearly distinguished from the power-stroke). At these negative x-values the cross-bridge detachment rate is appreciably accelerated [165,236] explaining a considerably higher velocity than predicted on the basis of the single-molecule data.

Other issues where there are discrepancies between single-molecule and muscle mechanics results or insufficient availability of experimental data are considered in the following section.

## 6. Top-Down and Bottom-Up Models

The interpretation in terms of cross-bridge function of experimental results from muscle or myofibril mechanical studies are, as indicated in Figure 6 above, based on averaging over a large spectrum of states and strains existing at any given time in a muscle or myofibril. The need for such averaging is not circumvented by synchronization efforts in transient perturbations because the perturbations do not remove the existing distributions of strains (cf. Figure 6a–c). For instance, at the end of a length step imposed on a muscle fiber, some cross-bridges may be in a state and strain where they rapidly detach whereas other cross-bridges are in states and strains where they are highly likely to undergo force-generating structural change(s). Such ensemble data are quantitatively treated using statistical models where force, stiffness, ATP turnover, etc. are calculated as average cross-bridge properties based on state probabilities [162,163,219]. The latter are calculated for each condition, e.g., by solving master equations in the state probabilities or by Monte-Carlo simulations [165,166,211,235,236,239] using a given set of parameter values that define transition rates, elastic properties and gross structural features such as power-stroke distances.

### 6.1. Challenges with Top-Down Models

Many models have derived parameter values from previous muscle mechanical experiments by fitting the model to data. These types of models, which we denote top-down models, suffer from ambiguities for several reasons. Primarily, development of cross-bridge models based on muscle mechanical data require appreciably more assumptions (educated guesses) than those defined on the basis of well-controlled single-molecule experiments. For instance, what are the properties of the elastic elements in cross-bridges and myofilaments, how large a fraction of the cross-bridges is attached, and what is their distribution between different states and strains? The existence of such a large number of uncertainties when interpreting experiments in terms of cross-bridge properties is reflected in a wide range of different models. These assume varying paths through the cycle [156,164,167,171,211,228,240] and various processes for force-generation, e.g., in relation to release of inorganic phosphate (Pi) and the number of force-generating structural changes.

A particular problem is that some models include states and transitions that cannot be readily identified in single-molecule experiments, structural data or solution biochemistry. As one illustrative example, most models assume several force-generating structural transitions (power-strokes), each corresponding to a few nm translations of myosin and actin relative each other. In contrast, single-molecule studies (in agreement with structural data) have only detected one major force-generating structural change of about 7–8 nm including a shorter step of ~1 nm, presumably associated with gating of the ADP release (see above). An intermediate position is taken by studies tracking quantum dots attached to myosin propelled actin filaments. In these studies, up to three sub-steps have been detected [241]. There are often well-founded reasons for the assumption of several sub-strokes in model studies based on muscle mechanics. First, one can argue that single-molecule studies, due to limited time resolution or other complications, have missed one or several sub-strokes. Second, (cf. [169]) there are theoretical arguments that it would be difficult to achieve the high forces of muscle with only one step if the force-generating process is of the Kramers type. With the current estimate of the cross-bridge stiffness, thermally driven diffusion of a nanoscale myosin head domain, typical of a Kramers process, would be too slow if it occurs in just a single step, to explain the high average force of muscle. However, again, as emphasized above, other authors have proposed that force-generation may occur without such large-scale diffusion [162,164], effectively being an Eyring process (cf. [213]). In the latter process there are different fundamental limitations than those applying to a Kramers processes because the diffusion occurs over very short distance (sub-nm) associated with a local chemical change followed by relaxation of the entire structure into a new equilibrium conformation. A third reason for assuming that there is more than one force-generating step, fundamental issues aside, is based on a fitting procedure that gives the best fit to experimental data with two sub-strokes [167]. However, also in the latter case, there may be alternative explanations. For instance, the need for two steps may be removed if the cross-bridge stiffness is somewhat lower than assumed and different results may be obtained if other details of the model are modified.

### 6.2. Bottom-Up Models to the Rescue?

An alternative to the top-down modelling considered above is the bottom up approach where the parameter values are derived from single-molecule data and ultrastructural studies (aided by biochemical analysis). This would avoid uncertainties in the parameter values and model structure due to averaging and effects of emergent properties. However, it requires that the relevant information can be achieved reliably and with sufficient detail. Important parameter values include the number of metastable states, the power-stroke distance, the force-dependent inter-state transitions and the parameter values defining the cross-bridge force-extension relationship. As outlined above, optical tweezers-based studies aided by biochemical solution kinetics can, in principle, provide all required characteristics to define such a mechanokinetic models (e.g., [165,220,236]). These parameter values and characteristics may now be plugged into an ensemble model with kinetic scheme based on biochemical kinetics and gross structural features consistent with both the single-molecule mechanics and ultrastructural studies. If this model can simulate muscle and myofibril behavior without assuming cooperative or other emergent phenomena this corroborates the idea that muscle mechanics can be fully explained based on single-molecule properties.

In partial support of the latter idea (however, see complications below), it was found recently that a simple model [165] accounts for a wide range of experimental data from single molecules over in vitro ensemble data (in vitro motility assays) to myofibrils and muscle. This model was largely defined on the basis of biochemical solution kinetics and single-molecule mechanics where the latter data grossly defined cross-bridge stiffness and approximate positions of the free energy of three attached states. Thereby, the model is largely of the bottom-up type as defined above. However, the positions of the free energy minima and the free energy levels at the minima were fine-tuned based on previous fittings to force-velocity relationships [164,221]. The need for this minimal correction may reflect uncertainties in the exact parameter values from single-molecule data (see above) or failures to completely mimic the in vivo environmental conditions in the in vitro experiments.

One further deviation from the bottom up approach that was necessary to impose in defining the model [165] was the exact force-dependence of rate constants for cross-bridge detachment from post-power stroke states. For negative cross-bridge strains it was necessary to assume faster detachment rate than suggested by single-molecule data [64] to account for the high maximum velocity of shortening of muscle. In contrast, for positive cross-bridge strain it was necessary to assume lower detachment rate to account for the slow rate of relaxation after an isometric tetanus. One possible reason for these discrepancies could be that one-headed myosin sub-fragment 1 was used in the single-molecule studies that gave the quantitative relationship between force and rate constants. On the other hand, reduced strain dependence of the cross-bridge detachment at negative x (corresponding to lower value of the rate constant), in agreement with the optical tweezers data [64], may be consistent with the observed high velocity [221] if the cross-bridge elasticity is non-linear as suggested by other single-molecule studies [67] (see further below).

When the model [165] was fully defined with correction of the bottom up data as described above, it was successful in accounting for a range of independent experimental findings, including myofibril and muscle experiments such as the rate of rise of isometric force and the force-velocity relationship. However, challenges were noted related to the relationship between [MgATP] and sliding velocity and the response of active muscle to stretches.

### 6.3. Bottom-Up Models and Non-Linear Cross-Bridge Elasticity

With regard to the velocity vs. [MgATP] relationship, the (mainly bottom-up) model [165] predicted a rectangular hyperbolic relationship in accordance with experiments. However, the [MgATP] for half maximal velocity, K_M_^v^, was appreciably lower in the model than in the experiments. This was also found in a previous study, interpreting in vitro motility assay data at different [MgATP] in terms of a somewhat simpler model [221]. Importantly as pointed out by Persson et al. [221] the K_M_^v^ in their in vitro motility assay data is likely similar to K_M_^v^ in muscle fibers at the same temperature (cf. [242]) suggesting that K_M_^v^ value predicted by the bottom-up defined model would be low compared to muscle fiber data. However, interestingly, it was found that the K_M_^v^ in the model was increased to that seen in muscle cells if the cross-bridge elasticity was assigned a non-linearity similar to that suggested by single-molecule studies on full length myosin in rigor [67]. The latter studies [67] showed several-fold lower stiffness in the drag-stroke region for a cross-bridge (with negative strain; x < 0 in Figure 6) than in the positively strained region (x > 0 in Figure 6) consistent with a bending of the lever-arm region for positive cross-bridge strains but buckling of the sub-fragment 2 domain of myosin in the negative strain region (see also [243]).

The change from linear to non-linear cross-bridge elasticity in the model [221], allowing reproduction of the experimental value of K_M_^v^, led to overestimation of the maximum sliding velocity. This complication was, however, readily amended by eliminating the force-dependence of the detachment rate constant from the post-power stroke state, fixing it to the level at zero elastic strain. This is also in better agreement with the single-molecule data [64]. Thus, the introduction of non-linear elasticity similar to that observed by Kaya and Higuchi [67] facilitates the fit of several aspects of muscle mechanics based on bottom-up model definition. Finally, as pointed out in [106] the non-linearity of the cross-bridge elasticity proposed in [67] is consistent with a drag stroke distance that is appreciably longer than the power-stroke distance during unloaded shortening of muscle [244]. This idea may be compatible with the X-ray interference data [199,225] suggesting a short (~10 nm) working stroke also at high sliding velocities if these data report the myosin sub-fragment 1 conformation without being appreciably affected by buckling of sub-fragment 2.

Despite the above arguments, there is no consensus as to whether the cross-bridge elasticity is indeed non-linear in the muscle cell. Some authors have presented evidence for non-linear elasticity in muscle cells under rigor conditions [198,222] and in active contraction [167,194]. However, it is not clear whether this non-linearity is an artefact, e.g., due to slackening of filaments [198,245], or whether it is attributed to the myofilaments or to the cross-bridges [222]. Additionally, some other authors have, on the basis of their experimental findings [199,245], argued strongly against any non-linearity whether in the myofilaments or in the cross-bridges [227,245,246]. Rather, it is claimed in these studies that a perceived non-linearity in some experiments is attributed to a parallel elastic element of importance only at low force levels [227,246]. However, the issue is by no means settled [192,212]. To achieve this as soon as possible and once and for all is of utmost importance. First, if there is a true difference between single-molecule results and results from muscle fibers it is of interest to understand the structural basis for it, e.g., whether it is related to the ordered 3D arrangement in the muscle cell or the presence of accessory proteins such as MyBPC. Second, it is important to clarify the situation in a muscle cell both with regard to the cross-bridge elasticity and the myofilament elasticity because the interpretations of a range of experimental results are strongly affected by the actual assumption made [106,244,247]. Finally, non-linearities in the cross-bridges and/or the myofilaments may have several fundamentally important roles, e.g., increased efficiency of contraction under certain conditions [106,221].

### 6.4. Jumping Cross-Bridges, Mechanosensing and Two Heads

In the recent model of Månsson [165] (see also [210]) it was found necessary to introduce a new type of transitions (cf. [156]) during stretch to allow instantaneous “jumps” from a pre-power-stroke state of cross-bridges at one site of actin to a pre-power-stroke state on the next myosin-binding site towards the pointed end of actin. Possible correspondence to these events in single-molecule studies are the observed sequence of up to five 5.3 nm displacements with the consumption of one ATP molecule by a single myosin head immobilized on a scanning probe [248]. Possibly the arrangement with attachment of the myosin motor domain to the scanning probe and the attachment of actin filaments to a surface could have promoted the jumping behavior. One reason might be increased local actin concentration (cf. [249]) seen by each myosin head, possibly to a value close to that in the myofilament lattice of muscle (>1 mM) [163,250]. Whether there is correspondence between the findings of Kitamura et al. [248] and the proposed jumping transitions during stretch, and possibly during shortening [152,251], it was impossible without such transitions to account for the peak force and some degree of maintenance of this force level during the stretch. However, it was pointed out [165] that the treatment of the stretch response was tentative due to ambiguous evidence in the literature. Some authors have thus suggested that the high force during stretch is primarily associated with attachment of more cross-bridges compared to isometric contraction without any appreciable increase in the average cross-bridge strain [154,245]. This mechanism was proposed, based on X-ray interference data [154], to be attributed to attachment of the second myosin head in a pair to actin during stretch with the capacity for up to two-fold increase in force without increase in average strain. On the other hand, Nocella et al. [158] presented evidence, based on stiffness measurements, suggesting only minor increase in the cross-bridge number during stretch. Instead, their results suggested that the largest fraction of the force enhancement during stretch is attributed to increase in the average cross-bridge strain. This idea is consistent with the model in [165]. The observed increase in stiffness during the stretch in the study of Nocella et al. [158] was attributed to non-linearity of the myofilament elasticity.

In addition to the cross-bridge mechanisms considered above there are other factors that are likely to affect the stretch response to appreciable degrees under certain conditions: These involve the large elastic protein titin as well as emergent phenomena [181,252,253]. It is now well documented in muscle fibers [252,254] and myofibrils [181] that titin has Ca^2+^-binding sites and that the molecule increase its stiffness during activation, which contributes to the increase in force observed during stretch at long sarcomere lengths.

In addition to roles during stretch, sequential action of the two heads of a given myosin molecule [164] as well as jumping of one head between neighboring sites on actin [156] have also been proposed to explain other phenomena. Of particular importance in this regard is the high power-output of skeletal muscle fibers when shortening against intermediate to high loads. Earlier modelling studies have suggested that the high power output is otherwise not consistent with a rather slow rate of rise of isometric force [156,161,164,255]. As an alternative mechanism, it has also recently been proposed that more cross-bridges are available for binding to actin during shortening at intermediate/high loads than during shortening against low loads and during the early rise of isometric force due to a mechanosensing effect [256]. The latter effect was first demonstrated by a combination of X-ray diffraction and mechanical experiments during the rise of tension after shortening in living frog muscle fibers [120]. As suggested in the same study and a more recent paper [119], the effect seems to be associated with the parking of the myosin heads on the thick filament backbone (presumably in a state analogous to the super-relaxed state) [40,41] at low tension in the filament. With increased tension, structural changes in the thick filament, e.g., related to MyBPC and/or titin [119,120], cause an increasing number of the myosin heads to be detached from the surface of the thick filaments thus becoming available for actin interactions. When they swing out towards the actin filament they contribute to tension development explaining an accelerating rate of rise of isometric force and possibly a higher power-output during shortening against intermediate to high loads. However, recent models [165] (and particularly [210]) seem to account for the high power-output alongside a rather slow rate of rise of isometric force without the need for any of the above complicating mechanisms such as jumping between sites, sequential actions of two heads or contribution from the mechanosensing mechanism. Nevertheless, further studies are needed to clarify these issues.

The role of the two heads of myosin II in muscle is a long-standing issue (cf. [257]) that has not yet been full clarified [212]. Although ideas of sequential actions of the two heads have been considered to account for the high maximum power output in muscle contraction [79,164,255] or the response to stretch of active muscle [154,245] (see above), similar sequential actions have been difficult to corroborate in single-molecule studies using two-headed myosin. A switch from one-headed to two-headed myosins in such studies has been associated with a doubling of the working stroke distance from about 5 to 10 nm [104]. This has generally not been interpreted as the sum of two working strokes but rather as a favorable positioning of one head by the other so that the properly positioned head can execute its full working stroke [104,258]. However, the possibility also exists that the longer observed working stroke with two-headed myosin is attributed to release of geometrical constraints associated with adsorption of a part of the single-headed myosin, rather inflexibly, to an artificial surface. One reason for the failure to observe true sequential head-operations in single-molecule studies may be that the evidence suggesting two-headed operation in muscle cells has other foundations. This could be due to the strong model dependence in the analysis of muscle fiber data (see above and [259]). Another possible basis for the difference between single-molecule studies and muscle results is the geometrical constraints imposed by the myofilament lattice in muscle. First, the close proximity of the thin and thick filaments in muscle (12–13 nm surface to surface) [260,261] may increase the effective actin concentration [163,250] with increased likelihood of attachment to a neighboring site, particularly if the second head is appropriately positioned by the power-stroke of the first head. In this connection, it is of interest to note that a given myosin head, when fixed in position relative to an actin filament in optical tweezers studies, can bind 3 neighboring actin sites along the filament without appreciable steric hindrance as the filament is translated past the myosin head [62]. Second, in contrast to the situation in single-molecule studies the two heads of a given myosin molecule may, at least under rigor conditions, bind two neighboring actin filaments simultaneously [207].

### 6.5. Roles of Accessory Proteins and Non-Uniform Sarcomere Behavior in Muscle Contraction in Health and Disease

The presence of several accessory proteins in muscle cells and myofibrils such as troponin, tropomyosin, titin and MyBPC, may also form the basis for differences between single-molecule mechanics and muscle cells. This was considered above in the section “Challenges in single-molecule mechanics” where we particularly emphasize the importance of these proteins in studies of diseases and drug effects. An additional issue that is pertinent to consider is effects of non-uniform sarcomere properties along a muscle cell [262,263] or myofibril [264]. These issues may, not the least be important to consider in future studies of cardiomyopathies and other diseases [265,266,267,268,269].

## 7. Conclusions and Perspectives

First of all, despite the differences and various complications associated with both single-molecule studies and muscle mechanics (geometry, surface-attachment, presence or absence of accessory proteins, order on different levels and averaging effects) the two types of studies give surprisingly consistent information about cross-bridge function. These findings support the general notion that any cooperative and emergent effects due to the hierarchical order and the presence of various accessory proteins in muscle have only minor modulatory effects on contractile function. Models such as those proposed in [165] thus give a good description of muscle function using parameter values from single-molecule data and biochemical kinetics. However, there are remaining uncertainties that need to be clarified before full understanding is possible:

1. Are there any sub-strokes in the power-stroke and is force-generation a Kramers or an Eyring process? Furthermore, related, the maximum power-stroke distance for which a single step is possible is about 9 nm (assuming an Eyring process)? Thus, a 9 nm working stroke with cross-bridge stiffness of 2 pN/nm corresponds to a maximum isometric cross-bridge force of less than 20 pN and maximum power of 81 pNnm ≈ 20 k_B_T, less than the free energy of ATP turnover under physiological conditions.
2. What are the characteristics and magnitude of the cross-bridge elasticity in muscle cells? Is the stiffness about 2 pN/nm or higher at positive strain and is the elasticity non-linear as proposed by single-molecule studies [67]? Furthermore, what is the characteristic of the myofilament elasticity in muscle cells?
3. What is the role of the two myosin heads? Is one of the heads just guiding the other to its binding site as suggested by single-molecule studies or can the ordered geometry of the myofilament lattice cause the heads to work in sequence?
4. Can the high maximum power-output of muscle be explained based on bottom-up defined models without assuming cooperative effects (e.g., between two heads), altered kinetics in an ordered ensemble (e.g., jumping between sites) or effects of mechanosensing in the thick filaments?
5. Can force-dependent rates obtained in optical tweezers studies predict the contractile behavior of muscle, for instance if the cross-bridge elasticity in muscle cells is non-linear as in [67]? Further model studies along these lines are of interest but also single-molecule studies to elucidate the force-dependence of rate constants in full length myosin.
6. What is the main cross-bridge dependent mechanism of the force-enhancement during stretch? Are the two heads important or is there jumping between neighboring sites, possibly related to stretch induced changes in actin with strongly enhanced affinity at neighboring sites. For further insight, it would be of interest to supplement the muscle mechanics studies with single-molecule experiments. To the best of our knowledge such studies have not yet been performed.
7. Are the modulatory roles of accessory proteins of increasing importance in diseases, e.g., as suggested for possible roles of MyBPC or titin in controlling the sequestered myosin head motif and also for mutations in RLC and other regulatory proteins such as troponin and tropomyosin. Further, are emergent phenomena enhanced due to increased sarcomere non-uniformities and mechanical instabilities in sarcomere myopathies?
